# Associations between DEET, Organophosphorus Insecticides, and Handgrip Strength in Diabetes: An NHANES Analysis

**DOI:** 10.3390/biomedicines12071461

**Published:** 2024-07-01

**Authors:** Chi-Feng Liu, Li-Wei Chien

**Affiliations:** 1 School of Nursing, National Taipei University of Nursing and Health Science, Taipei 112, Taiwan; 2 Department of Obstetrics and Gynecology, School of Medicine, College of Medicine, Taipei Medical University, Taipei 110, Taiwan; chienwei@tmu.edu.tw; 3 Department of Obstetrics and Gynecology, Taipei Medical University Hospital, Taipei 110, Taiwan

**Keywords:** diabetes mellitus (DM), handgrip strength, insecticides, muscle health, sarcopenia, National Health and Nutrition Examination Survey (NHANES), pesticide

## Abstract

Introduction: Sarcopenia and diabetes mellitus (DM) have been shown to be related. It has been demonstrated that pesticides/insecticides are linked to various health issues, including DM. This study investigated the relationships between exposure to pesticides/insecticides and muscle strength among community-dwelling DM patients in a national sample of the United States (US). Methods: Data from the 2011–2012 and 2013–2014 U.S. National Health and Nutrition Examination Survey (NHANES) on people aged 20 years with diabetes were retrieved. A digital dynamometer was used to quantify handgrip strength, and urine pesticide concentrations were determined through laboratory testing. Regression models were used to investigate the relationship between pesticide/insecticide exposure and handgrip strength. Results: After weighting, the data from 412 NHANES participants represented 6,696,865 U.S. inhabitants. The mean age of the participants was 58.8 years. High para-nitrophenol levels (tertile 3 vs. tertile 1) were shown to be associated with lower handgrip strength in both males (aBeta = −7.25, 95% CI: −11.25, −3.25) and females (aBeta = −3.73, 95% CI: −6.89, −0.56). Further, females with elevated 2-isopropyl−4-methyl-pyrimidinol had decreased handgrip strength. Desethyl hydroxy N, N-diethyl-m-toluamide (DEET) was inversely related to handgrip strength in men aged ≥60 years. DEET acid and para-nitrophenol were inversely correlated to handgrip strength in women over 60 years. Conclusions: This study has linked certain pesticides/insecticides to decreased muscle strength in people with diabetes. Para-nitrophenol, in particular, is negatively related to muscular strength in both males and females, and 2-isopropyl-4-methyl-pyrimidinol is inversely related to muscle strength in females.

## 1. Introduction

Diabetes mellitus is a prominent contributor to global morbidity and mortality, affecting individuals irrespective of their nationality, age, or sex. In 2021, it was estimated that 529 million people worldwide had diabetes, with a prevalence of 6.1% [1]. By 2050, the number is projected to increase to over 1.31 billion, and 44% of countries will surpass a 10% prevalence rate [1,2,3]. Diabetes often leads to a range of health complications, such as heart disease, chronic kidney disease, liver disease, perivascular disease, nerve damage, and various conditions affecting the feet, oral health, vision, hearing, and mental well-being [4,5].

In recent years, there has been growing attention directed toward the relationship between diabetes and sarcopenia. Sarcopenia entails a reduction in muscle mass, muscle strength, and physical function, and is primarily associated with advanced age [6]. It can profoundly affect the well-being of older individuals and is associated with the occurrence of falls, fractures, mortality, and various other adverse events [6]. Recent studies have indicated that diabetes is a contributing factor to the development of sarcopenia [7,8,9]. Insulin resistance in skeletal muscles leads to reduced utilization of glucose and decreased protein synthesis, thereby intensifying both insulin resistance and muscle loss, creating a vicious cycle [7,8,9]. Studies have indicated that nearly 70% of adults with diabetes have difficulty performing routine physical tasks, with marked limitations on lower limb mobility [7,8,9,10]. A very recent Mendelian randomization study that investigated sarcopenia-related traits using a genome-wide association analysis found a possible bidirectional, causal relationship between type 2 diabetes mellitus (T2DM) and handgrip strength and walking pace [11]. Furthermore, impaired muscle health has been associated with decreased survival in patients with T2DM [12,13]. Consequently, it is important to determine factors that affect muscle health in people with diabetes.

Pesticides are used worldwide to manage pests that threaten food crops, animals, and humans. However, their use can lead to the contamination of food and water systems, potentially endangering human health through the consumption of contaminated food and water, or occupational exposure (e.g., farmers) [14]. Pesticide exposure has been associated with various health conditions, including Hodgkin disease (HD), non-Hodgkin lymphoma (NHL), Parkinson’s disease, and endocrine, respiratory, and reproductive disorders [15,16]. Recent epidemiological research has also linked pesticide exposure to metabolic conditions, including diabetes [17]. Although the molecular processes have not been fully elucidated, studies have indicated that pesticides have an adverse impact on handgrip strength and skeletal muscle mass, especially among overweight and obese individuals [18].

Pesticides are generally classified by target (fungicides, insecticides, herbicides, rodenticides) and composition (organic or inorganic). Organic pesticides have diverse chemical structures and include chlorohydrocarbon-, organophosphorus-, and carbamate-based chemicals [19]. N, N-diethyl-m-toluamide (DEET) is a primary ingredient in commonly used insect repellents and has exceptional effectiveness in warding off biting insects like mosquitoes and ticks [20]. However, there has been growing concern and debate regarding the safety of DEET. Research indicates that both chronic and acute DEET exposure can lead to adverse effects in both children and adults, such as neurological and cardiovascular disorders [21,22]. Moreover, pyrethroids, herbicides, and organophosphorus insecticide metabolites have all been linked to a range of negative health effects [21,23,24].

To date, there have been no studies published in the medical literature exploring the possible relationship between pesticide exposure and muscle strength in people with diabetes. Therefore, this study aimed to evaluate the association between pesticide exposure and muscle strength in individuals with diabetes, using data from the United States (U.S.) National Health and Nutrition Examination Survey (NHANES).

## 2. Materials and Methods

### 2.1. Study Design and Ethical Considerations

The present study analyzed data from the NHANES dataset, which is collected by the Centers for Disease Control and Prevention (CDC), National Center for Health Statistics (NCHS) in the United States. The NHANES survey is designed to cover the socioeconomic, health, behavioral, and nutritional status of adults and children. Participants in the NHANES are invited for an extensive examination in a mobile examination center (MEC), including a physical examination, specialized measurements, and laboratory tests after completing a household interview. These evaluations and data collections are considered reliable and multidimensional, from which national estimates can be derived. Details of the NHANES database and data collection can be found at: http://www.cdc.gov/nchs/nhanes/ (accessed on 1 August 2023).

As this study was a secondary analysis of data contained in a de-identified public database, it did not directly involve the participants. The NHANES receives approval from the NCHS Research Ethics Review Board, and all survey participants provide informed consent. All NHANES data are de-identified, and the data remain anonymous throughout any data analysis. Accordingly, no additional ethical approval or informed consent was required for the current study. The aforementioned NCHS approval can be obtained from the NHANES website: https://www.cdc.gov/nchs/nhanes/irba98.htm (accessed on 1 August 2023).

### 2.2. Study Population

Data from 2 NHANES cycles were used in this study: NHANES 2011–2012 and NHANES 2013–2014. These cycles were chosen because the NHANES collected different variables across study cycles to manage budget constraints, and these two cycles are the only ones that include available data on grip strength. The inclusion criteria were adults aged ≥ 20 years old with diabetes. The diagnosis of diabetes was based on the American Diabetes Association criteria, as identified by any one of the following recorded in the participants’ NHANES file: (1) self-reporting of a doctor’s diagnosis; (2) currently using oral hypoglycemic medication or insulin; (3) a HbA1c level ≥ 6.5%; (4) fasting glucose ≥ 126 mg/dL; or (5) a 2 h oral glucose tolerance test (OGTT) result ≥ 200 mg/dL. The exclusion criteria were: (1) pregnancy; (2) end-stage renal disease (ESRD) defined as an estimated glomerular filtration rate (eGFR) < 15 mL/min/1.73 m^2^; or (3) missing information on handgrip strength, study variables, or urine pesticide metabolite levels.

### 2.3. Muscle Strength

Muscle strength was measured using a handgrip dynamometer. A trained examiner explained and demonstrated the procedure, adjusted the dynamometer grip size, and conducted a practice trial. The participant then squeezed the dynamometer firmly with each hand, alternating hands, for three trials per hand with a 60 s rest between measurements. The specific details of the grip test can be found on the NHANES website: https://wwwn.cdc.gov/Nchs/Nhanes/2011-2012/MGX_G.htm (accessed on 1 August 2023).

The combined grip strength was used for the analysis in this study and is determined by the sum of the highest reading from each hand expressed in kilograms, as defined in prior studies [25,26].

### 2.4. Urine Pesticide Concentrations

#### 2.4.1. DEET

Exposure to DEET was determined by measuring the concentration of DEET and 2 of its metabolites, N, N-diethyl-3-hydroxymethylbenzamide (DHMB), and 3-diethyl-carbamoyl benzoic acid (DCBA), in a 100 µL sample of urine. Concentrations were measured using solid-phase extraction combined with high-performance liquid chromatography–tandem mass spectrometry (SPE–HPLC–MS/MS). The laboratory protocol is available at https://wwwn.cdc.gov/Nchs/Nhanes/2011-2012/DEET_G.htm (accessed on 1 August 2023).

#### 2.4.2. Organophosphorus Insecticide Metabolites, Synthetic Pyrethroid Metabolites, and Herbicides

The NHANES analysis included the measurement of urine concentrations of 2 organophosphorus insecticide metabolites, namely 2-isopropyl-4-methyl-pyrimidiol, and para-nitrophenol; 3 synthetic pyrethroid metabolites, namely 3-phenoxybenzoic acid, 4-fluoro-3-phenoxybenzoic acid, and trans-3-(2,2-dichlorovinyl)-2,2-dimethyl-cyclopropane-1-carboxylic acid; and 1 herbicide, namely 2,4-dichlorophenoxyacetic acid. The testing protocol is available at https://wwwn.cdc.gov/Nchs/Nhanes/2011-2012/UPHOPM_G.htm (accessed on 1 August 2023).

In cases where the analytical results were below the lower limit of detection (LLOD), an imputed fill value was assigned to the analyte results field. This imputed value was calculated as the LLOD divided by the square root of 2.

### 2.5. Covariates

The demographic data collected included age, sex, race, poverty-to-income ratio, and education level, and were obtained by questionnaires through in-person home interviews conducted by trained interviewers using the Family and Sample Person Demographics questionnaires and the Computer-Assisted Personal Interviewing (CAPI) system (Confirmit Corp., New York, NY, USA). The collected data were weighted according to the NHANES protocol.

To estimate physical activity, the product of the weekly time spent on each type of exercise activity reported by the participant multiplied by the metabolic equivalent of task (MET) value for that activity was summed, yielding a MET-h index. One MET is the energy expenditure of 1 kcal/kg body weight per hour. A MET-h/week ≥ 48.1 in men and >27.1 in women is considered vigorous-intensity physical activity. A MET-h/week 8.1 to ≤48 in men and 3.1 to ≤27 in women is defined as moderate intensity. A MET-h/week ≤ 8 in men or ≤3 in women suggests low-intensity activity.

Body mass index (BMI) was obtained from the NHANES MEC examination measurements, and calculated as body weight (kilograms) divided by height (meters squared). Body weight was measured using an electronic load cell scale, and standing height was measured with a fixed stadiometer.

Smoking status was classified as non-smoker (lifetime smoking < 100 cigarettes), former smoker (lifetime smoking > 100 cigarettes, but not currently smoking), and current smoker (lifetime smoking > 100 cigarettes and currently smoking).

Hypertension was defined as a “yes” to being diagnosed with high blood pressure on 2 or more visits or prescribed medication and also diagnosed with an average systolic BP ≥ 140 mm Hg or diastolic BP ≥ 90 mm Hg from 3 consecutive measures.

Hyperlipidemia was defined as a “yes” to prescribed medication for cholesterol or serum total cholesterol level > 200 mg/dL.

Cardiovascular disease (CVD) was defined as a “yes” to prescribed medication for cholesterol or serum total cholesterol level > 200 mg/dL.

Chronic respiratory tract disease was defined as a “yes” response to being diagnosed with emphysema or chronic bronchitis by a healthcare professional.

GFR was estimated from a re-calibrated serum creatinine level using the 4-variable Modification of Diet in Renal Disease (MDRD) Study equation. The IDMS-traceable MDRD Study equation that uses standardized creatinine is eGFR = 175 × (standardized serum creatinine)^−1.154^ × (age)^−0.203^ × 0.742 (if the subject is a woman) × 1.212 (if the subject is black). The eGFR was reported as mL/min/1.73 m^2^. Chronic kidney disease (CKD) was defined as an eGFR < 60 mL/min/1.73 m^2^.

Cancer was defined as a “yes” response to being diagnosed with cancer or malignancy by a healthcare professional.

Arthritis was defined as a “yes” response to being diagnosed with arthritis by a healthcare professional.

Insulin dependence was defined as a “yes” response to currently taking insulin. Poor diabetes control was defined as HbA1c > 9.0%.

Energy and protein intake were determined through the 24 h dietary recall data from the NHANES.

### 2.6. Statistical Analysis

The NHANES uses a complex, multistage, probability sampling design to assure national representation, wherein the sampling weights (WTMEC2YR), pseudo-stratum (SDMVSTRA), and pseudo-cluster (SDMVPSU) provided by the NHANES are applied in all analyses, as guided by the NCHS. The weighted mean and standard error were presented for continuous variables; unweighted number and weighted proportion were presented for categorical variables. P-values for group comparisons were calculated using the PROC SURVEYFREQ and SURVEYREG for categorical and continuous data, respectively. Logistic regression models were performed with PROC SURVEYREG to identify the association between urine pesticide concentrations (independent variable) and muscle strength (dependent variable) in patients of different sexes. Multivariable regression was adjusted for covariates with a *p*-value < 0.05 (Supplementary Table S1). For males, this included age (continuous), BMI, hypertension, CVD, CKD, arthritis, and energy intake (continuous), and for females, this included age (continuous), race, BMI, hypertension, CVD, CKD, cancer, arthritis, energy intake (continuous), and protein consumption (categorical). The detectable percentage of urinary levels of pesticide metabolites ≥ LOD, geometric means (GMs) with 95% CIs, and weighted tertiles of the metabolite concentrations are presented in Supplementary Table S2. Weighted proportions of concentrations above the LOD of these pesticide metabolites were >95%. In all analyses, a 2-sided value of *p* < 0.05 was regarded as statistically significant. All statistical analyses were performed using SAS statistical software version 9.4 (SAS Inc., Cary, NC, USA). 

## 3. Results

### 3.1. Study Population

**Figure 1:**
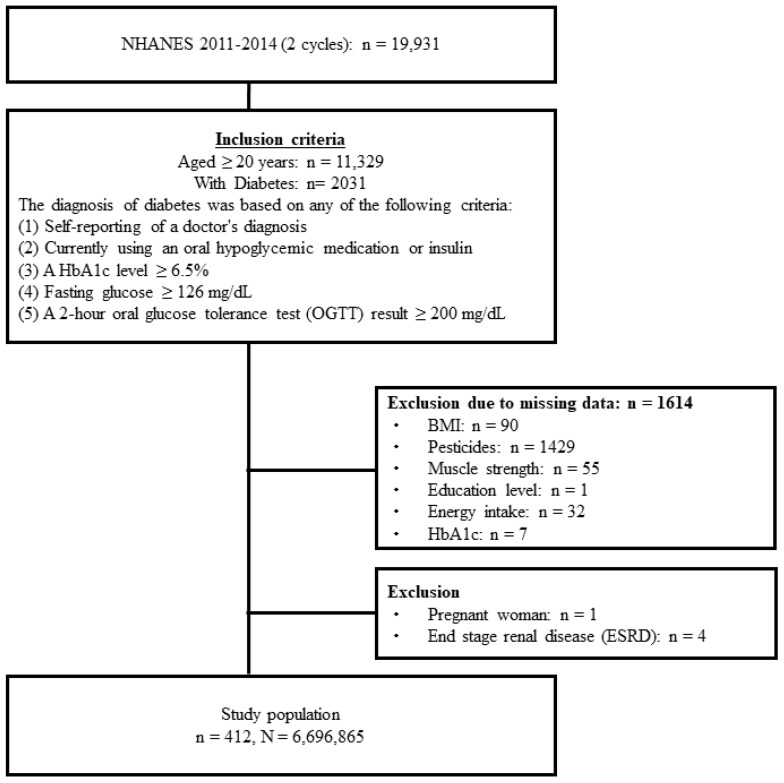
Flow diagram of participant inclusion.

A total of 19,931 participants were included in the two NHANES cycles examined in this study. Of these participants, 2031 persons ≥ 20 years of age had diabetes. Of these, 1614 participants with missing information on BMI (*n* = 90), urine pesticide levels (*n* = 1429), muscle strength (*n* = 55), education level (*n* = 1), energy intake (*n* = 32), and HbA1c (*n* = 7) were excluded. In addition, one pregnant woman and four participants with ESRD were excluded. Finally, 412 NHANES participants were included in this analysis. Using the weight values provided by the NHANES, this study sample could be extrapolated to represent 6,696,865 individuals in the entire United States, after weighting (Figure 1).

### 3.2. Characteristics of the Study Population

The mean age of the participants was 58.8 ± 0.9 years, most of them were White (64.8%), 81.4% of the participants had a low-intensity physical activity level, 50.1% were never smokers, and the most common comorbidity was hyperlipidemia (76.8%) (Table 1 and Table S1).

Supplementary Table S2 shows the distribution of urine pesticide levels (ng/mL creatinine), including the limit of detection (≥LOD), geometric means (GMs) with 95% CIs, and weighted tertiles (Supplementary Table S2).

### 3.3. Associations between Urine Pesticide Levels and Muscle Strength

The associations between urine pesticide levels and grip strength are summarized in Table 2. After adjusting for relevant confounders, a high para-nitrophenol level (tertile 3 vs. tertile 1) was significantly associated with decreased muscle strength in males (adjusted β [aBeta] = −7.25). High para-nitrophenol was also associated with significantly decreased muscle strength in females (tertial 2 vs. tertial 1: aBeta = −4.01; tertial 3 vs. tertial 1: aBeta = −3.73; as a continuous variable: aBeta = −1.87).

After adjustments, the high 2-isopropyl-4-methyl-pyrimidinol level was significantly associated with decreased muscle strength in females (tertial 3 vs. tertial 1: aBeta = −3.85; as a continuous variable: aBeta = −2.07) (Table 2).

The full analytic models of the associations between study variables and muscle strength are summarized in Supplementary Table S3.

We also stratified the analysis according to physical activity levels (vigorous/moderate intensity and low intensity). The results are shown in Supplementary Tables S4 and S5. We found that regardless of the intensity of physical activity, the pesticide/insecticide levels were significantly associated with decreased muscle strength.

We further checked the associations between study variables and muscle strength for participants without DM under the same conditions. These results are shown in Supplementary Table S6.

### 3.4. Associations between Urine Pesticide Levels and Muscle Strength Stratified by Age

**Figure 2:**
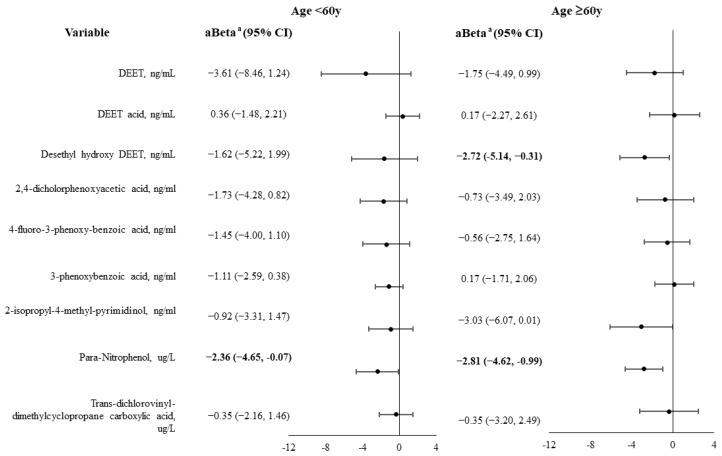
Associations between urine pesticide levels and muscle strength in males with diabetes, stratified by age group. Variables with a value of *p* < 0.05 are shown in bold. ^a^ Adjusted for related variables with a value of *p* < 0.05 in Supplementary Table S1, including body mass index, hypertension, chronic kidney disease, cardiovascular disease, arthritis, and energy intake (continuous).

**Figure 3:**
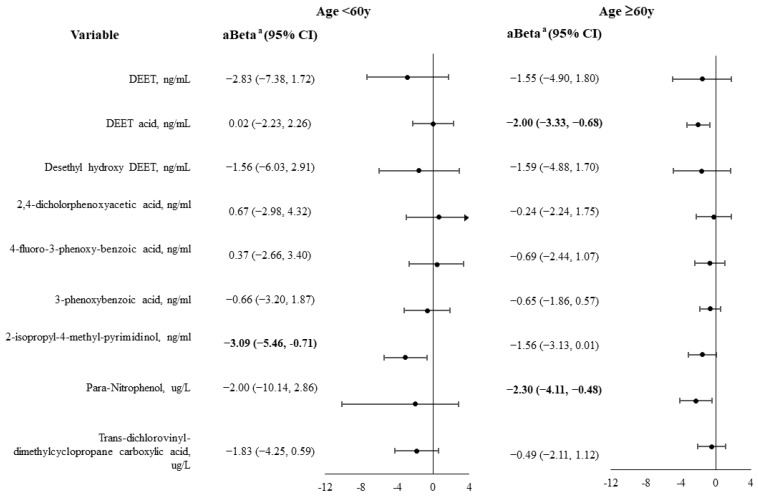
Associations between urine pesticide levels and muscle strength in females with diabetes, stratified by age group. Variables with a value of *p* < 0.05 are shown in bold. ^a^ Adjusted for related variables with a value of *p* < 0.05 in Supplementary Table S1, including race, body mass index, hypertension, chronic kidney disease, cardiovascular disease, cancer, arthritis, energy intake (continuous), and protein consumption (categorical).

The results of the stratified analysis according to age are shown in Figure 2 and Figure 3. In males, para-nitrophenol was significantly associated with decreased muscle strength regardless of age. In men ≥ 60 years old, the desethyl hydroxy DEET level was significantly and inversely associated with muscle strength (aBeta = −2.72) (Figure 2).

In women <60 years old, 2-isopropyl−4-methyl-pyrimidinol was significantly and inversely associated with muscle strength (aBeta = −3.09). In women ≥60 years old, DEET acid and para-nitrophenol were significantly and inversely associated with muscle strength (aBeta = −2.00; aBeta = −2.30, respectively) (Figure 3).

## 4. Discussion

This NHANES-based study of adults with diabetes found that para-nitrophenol levels were inversely associated with muscle strength in males and females. Additionally, 2-isopropyl-4-methyl-pyrimidinol levels were inversely associated with muscle strength in females. Age-stratified analysis revealed varying degrees of the correlations between urine pesticide levels and muscle strength in different age groups, but the results generally suggested that higher exposures to DEET, 2-isopropyl-4-methyl-pyrimidinol, and para-nitrophenol are independently associated with decreased muscle strength across age and sex categories. The supplemental analysis did not reveal such association between these pesticide/insecticide and muscle strength among individuals without diabetes.

On the other hand, researchers have indicated bidirectional causal links between T2DM and handgrip strength [11]. Additionally, studies have emphasized the connection between compromised muscle health and a poorer prognosis in individuals with T2DM [12,13]. Consequently, preventing impaired muscle health and searching for its potential predictors are crucial concerns in the management of diabetes. Moreover, there have been findings demonstrating associations between pyrethroid exposure and skeletal muscle strength and mass in the general population [18]. Collectively, these findings inspired us to investigate the impact of pesticides on muscle health in diabetic patients.

In this study, we examined the effect of several types of pesticides on the muscle strength of people with diabetes. While the existing literature lacks specific studies that have focused on such associations, several studies have noted relationships between pesticide exposure and diabetes and various adverse health conditions [15,27,28,29,30,31,32]. One of the most well-known relationships is between organophosphorus pesticide exposure and new-onset diabetes, which is postulated to be due to pancreatic beta cell damage and/or decreased elimination of the chemical by hepatocytes due to a genetic polymorphism [15]. Organophosphorus exposure has also been linked to blood pressure dysregulation and hypertension [27], and alterations of sex hormone levels in males and females [32]. A recent NHANES analysis found that people with pyrethroid exposure had a 2.18-fold increased risk of developing diabetes [29]. As with organophosphorus compounds, pyrethroid exposure has been linked with alterations of sex steroid hormone levels in males and females [30]. Notably, a recent study found that exposure to environmental pollutants, including some pesticides, significantly increased the risk of women developing gestational diabetes mellitus during pregnancy [31]. A recent systematic review and meta-analysis also found that maternal pesticide exposure was associated with a significantly increased risk of preterm birth [28].

As one of the focal points of our study, DEET is one of the most common insect repellents and is very effective in reducing bites from insects like mosquitoes and ticks [20]. However, in recent years, the safety of DEET has become a topic of debate, and research suggests that acute and chronic exposure can lead to adverse health effects in children and adults [21,22,33]. However, studies have provided conflicting results, and have found relations between exposure and some markers of human health but not others. For example, some studies have linked DEET exposure with obesity in adults and hyperuricemia in adults [34,35]. On the other hand, an NHANES analysis found DEET exposure was associated with an increased risk of cardiovascular disease and coronary heart disease, but not with myocardial infarction, congestive heart failure, angina, or stroke [36]. Another NHANES analysis found no correlations between DEET exposure and C-reactive protein level, lymphocyte level, liver enzymes, or estimated glomerular filtration rate [37]. However, in the existing literature, there is no precedent for establishing a link between DEET and muscle health, regardless of components such as muscle strength, mass, or function. The results of the present study thus offer an essential initial association, warranting further comprehensive investigation and exploration of the underlying mechanisms in future research.

In this study, consistent adverse associations were observed between two metabolites of organophosphorus insecticides, namely para-nitrophenol and 2-isopropyl-4-methyl-pyrimidinol, and reduced muscle strength. Para-nitrophenol, which is a non-persistent pesticide metabolite, corresponds to the parent compound methyl parathion. It is worth noting that while parathion has been banned for residential and agricultural purposes in the United States since 2000, methyl parathion, along with diazinon, chlorpyrifos, and methyl chlorpyrifos, remains authorized for agricultural use. Consequently, metabolites of these insecticides continue to be easily detectable in the general population, indicating significant environmental exposures [27]. The prolonged persistence of these insecticides can be partly attributed to their chemical properties, making them highly lipid soluble and resistant to biodegradation in certain environments [22]. A previous NHANES study has indicated an association between para-nitrophenol and serum sex hormones [38]. Nonetheless, to date, no prior research has established a correlation between para-nitrophenol and impaired muscle health, making our study particularly innovative in this regard.

The influence of pesticides on muscle strength varies due to their distinct biochemical impacts on human health. For instance, organophosphorus pesticides inhibit acetylcholinesterase in the nervous system, leading to acetylcholine overactivity in the synapse and neuromuscular junction, which can impair neuromuscular transmission and muscle strength [15]. Pyrethroids, such as DDT, bind to voltage-gated sodium channels, preventing their transition from an activated to an inactivated state, potentially altering muscle function and strength [39]. Additionally, studies have shown that glyphosate inhibits cholinesterase activity in muscle tissues, which can impair muscle function and coordination [40]. These biochemical interactions might explain the differential impacts of various pesticides on muscle strength, underscoring the need for careful management of pesticide exposure to mitigate potential health risks.

The potential ways in which organophosphate insecticides may affect muscle strength are by influencing pathways controlled by microRNAs (miRNAs). MicroRNAs are powerful post-transcriptional regulators of gene expression in muscle and are detectable in the circulation. Disruption in miRNA expression can lead to muscle atrophy and functional loss [41]. Organophosphate insecticides have been observed to alter the expression of multiple miRNAs in both living organisms and laboratory settings [42]. Many of these miRNAs are linked to genes in cardiac, neural, and bone tissues, and myogenesis of skeletal muscle [43]. In addition, organophosphates, along with other types of pesticides, have been demonstrated to induce oxidative stress in different organ systems, potentially compromising their integrity [44,45]. Such oxidative stress may also have implications for the aging of muscle tissue [46]. Furthermore, miRNAs have been discovered to regulate inflammation, insulin resistance, and T2DM pathology. Elevated plasma concentrations of inflammatory mediators such as tumor necrosis factor α and interleukin-6 are linked to insulin resistance and T2DM. Changes in miRNA expression can affect these pathways, making miRNAs potential biomarkers for the diagnosis and progression of T2DM [47]. To substantiate the suggested biological mechanisms that underlie the observed associations, forthcoming experimental and epidemiological investigations should be conducted.

Our age-stratified analysis indicates that individuals aged 60 years and older show a more significant decline in muscle strength compared to younger age groups. Muscle fiber loss begins around age 50, with about 50% lost by age 80. This decline is accompanied by age-related inflammation, including decreased T and B cells, increased natural killer cells, and elevated levels of tumor necrosis factor-α (TNF-α), interleukin-6 (IL-6), and C-reactive protein (CRP), which may contribute to sarcopenia by activating the ubiquitin–proteasome system [48]. DM itself is an independent contributor to sarcopenia [49]. A bidirectional Mendelian randomization study further demonstrated that low handgrip strength can increase the risk of T2DM, while T2DM can further impair muscle health [50]. As such, our findings emphasize the critical need to address pesticide and insecticide exposures in this vulnerable population to prevent further deterioration of muscle health.

### Strengths and Limitations

This is the first study examining associations between urine pesticide levels and muscle strength in persons with diabetes. The results are based on a nationally representative database, and the findings are likely generalizable to the whole United States population. Demographic features, lifestyle factors, and chronic comorbidities were considered in the analyses. Specifically, our current analyses strive to minimize confounding. Firstly, we have meticulously considered and included all potential confounders that may affect the association between pesticides/insecticides and muscle strength. These potential confounders, which are documented risk factors for sarcopenia, include various chronic conditions such as hypertension, hyperlipidemia, cardiovascular disease, chronic obstructive pulmonary disease, chronic kidney disease, cancer, arthritis, and diabetes severity (indicated by insulin dependence and poor control, as reflected by HbA1c). Socio-environmental factors like the poverty–income ratio and education level were also considered. We also performed age-stratified analyses to determine associations in different age groups.

This study nevertheless has several limitations. First, muscle health encompasses various facets, including muscle mass, strength, and function. This study specifically concentrated on muscle strength. We did not include muscle mass in our analysis due to the limited availability of the NHANES muscle mass data, which was solely collected in the earlier cycles from 1999 to 2004 and those cycles did not include measurements of our primary study variable, pesticides. Further, the NHANES dataset, and consequently our analysis, did not distinguish between type 1 and type 2 diabetes. Diabetes duration was not included in the analysis due to substantial missing data, comprising more than one-third of the dataset. Despite our thorough approach, we acknowledge that there may still be other confounders not captured by the NHANES database or unrecognized confounders that were not collected. Another important issue is its cross-sectional nature, in which causal inferences cannot be made. Accordingly, such design also does not allow investigation of time-dependent muscle strength deterioration. Several comorbidities were identified through questionnaires, where inaccurate reporting or recall bias might influence the results. Additionally, although we have tried our best to include as many covariates as possible, there may be unknown confounders not collected by the NHANES survey that could impact muscle strength.

## 5. Conclusions

This study demonstrates a novel association between pesticide exposure and reduced muscle strength in adults with diabetes, particularly highlighting the negative impact of para-nitrophenol on muscle health in both sexes and 2-isopropyl-4-methyl-pyrimidinol levels in women. The findings indicate that higher exposures to these pesticides are independently associated with decreased muscle strength, underscoring the need for further research and targeted public health policies to mitigate these potential impacts. Future prospective investigations should focus on elucidating the underlying mechanisms and exploring the long-term consequences of pesticide exposure on muscle health in diabetic populations.

## Figures and Tables

**Table 1:** Characteristics of the study population.

	Total	Male	Female	
	N = 412	n = 211	n = 201	*p*
Muscle strength, kg	67.4 ± 1.4	83.0 ± 1.5	50.8 ± 1.1	**<0.001**
Age, years	58.8 ± 0.9	58.9 ± 1.1	58.7 ± 1.2	0.885
<50	99 (26.0)	50 (25.2)	49 (26.9)	0.302
50–59	83 (21.8)	41 (20.4)	42 (23.2)	
60–69	114 (26.1)	61 (30.7)	53 (21.1)	
70+	116 (26.1)	59 (23.7)	57 (28.7)	
Physical activity in leisure time METs-hours/week				
Vigorous intensity	7 (1.2)	5 (1.7)	2 (0.6)	**<0.001**
Moderate intensity	78 (17.4)	24 (7.4)	54 (27.9)	
Low intensity	327 (81.4)	182 (90.8)	145 (71.5)	
BMI, kg/m^2^				
Normal (<25)	50 (9.7)	25 (9.4)	25 (10.1)	0.980
Overweight (25–29.9)	131 (29.8)	74 (30.0)	57 (29.5)	
Obese (≥30.0)	231 (60.5)	112 (60.5)	119 (60.4)	
Smoking status				
Never	212 (50.1)	86 (37.1)	126 (63.9)	**<0.001**
Former	129 (32.5)	85 (43.1)	44 (21.3)	
Current	70 (17.4)	39 (19.8)	31 (14.8)	
Missing	1	1	0	
Major comorbidity				
Hypertension	290 (70.2)	142 (65.6)	148 (75.0)	0.113
Hyperlipidemia	312 (76.8)	152 (70.7)	160 (83.4)	**0.002**
CVD	106 (27.9)	60 (33.5)	46 (21.9)	0.051
COPD	38 (11.5)	11 (8.7)	27 (14.4)	0.275
CKD	85 (20.5)	41 (22.1)	44 (18.9)	0.568
Cancer	63 (19.5)	32 (22.7)	31 (16.1)	0.119
Arthritis	182 (42.8)	72 (32.4)	110 (53.8)	**0.002**
Energy intake, kcal/day	1990.3 ± 64.0	2294.6 ± 105.5	1667.2 ± 50.8	**<0.001**
Protein consumption, g/day	78.9 ± 2.6	90.9 ± 3.6	66.2 ± 3.0	**<0.001**
Poor control of T2DM	68 (16.5)	33 (15.5)	35 (17.5)	0.610
Insulin dependence	87 (21.7)	39 (19.4)	48 (24.2)	0.400

BMI, body mass index; CVD, cardiovascular disease; COPD, chronic obstructive pulmonary disease; CKD, chronic kidney disease; MET, metabolic equivalent task; T2DM, type 2 diabetes mellitus. Continuous variables are presented as mean ± standard error; categorical variables are presented as unweighted counts (weighted percentage). Variables with a value of *p* < 0.05 are shown in bold.

**Table 2:** Association between urine pesticide levels and muscle strength in adults with diabetes, stratified by sex.

	Muscle Strength, kg			
	Male ^a^		Female ^b^	
	aBeta (95% CI)	*p*	aBeta (95% CI)	*p*
DEET, ng/mL ^c^				
Tertile 2	1.30 (−7.71, 10.30)	0.771	−0.31 (−4.70, 4.08)	0.887
Tertile 3	−4.33 (−11.54, 2.87)	0.229	−1.23 (−5.42, 2.97)	0.555
Log-transformed	−2.22 (−5.74, 1.30)	0.207	−1.26 (−4.19, 1.66)	0.385
DEET acid, ng/mL ^c^				
Tertile 2	2.10 (−4.51, 8.72)	0.521	0.47 (−4.03, 4.97)	0.832
Tertile 3	2.24 (−1.79, 6.27)	0.266	−1.48 (−4.73, 1.78)	0.363
Log-transformed	0.05 (−0.93, 1.03)	0.921	−0.61 (−1.64, 0.43)	0.239
Desethyl hydroxy DEET, ng/mL ^c^				
Tertile 2	0.63 (−8.08, 9.34)	0.883	−0.50 (−5.32, 4.31)	0.833
Tertile 3	−4.64 (−12.14, 2.86)	0.216	−2.40 (−5.70, 0.90)	0.148
Log-transformed	−1.10 (−4.00, 1.79)	0.444	−1.81 (−4.31, 0.69)	0.151
2,4-dicholorphenoxyacetic acid, ng/mL ^c^				
Tertile 2	−0.95 (−7.28, 5.39)	0.762	−1.80 (−5.04, 1.44)	0.265
Tertile3	−1.60 (−6.10, 2.91)	0.475	−1.43 (−5.25, 2.39)	0.451
Log-transformed	0.25 (−1.79, 2.29)	0.802	0.08 (−1.86, 2.01)	0.936
4-fluoro-3-phenoxy-benzoic acid, ng/mL ^c^				
Tertile 2	1.03 (−7.57, 9.62)	0.809	−1.12 (−5.64, 3.40)	0.617
Tertile 3	−2.02 (−7.30, 3.26)	0.441	−0.66 (−5.29, 3.97)	0.773
Log-transformed	−0.46 (−2.59, 1.67)	0.661	−0.65 (−2.41, 1.10)	0.453
3-phenoxybenzoic acid, ng/mL ^c^				
Tertile 2	0.39 (−6.70, 7.48)	0.911	0.01 (−4.15, 4.17)	0.995
Tertile 3	−1.58 (−5.86, 2.71)	0.458	−0.80 (−3.06, 1.47)	0.478
Log-transformed	−0.76 (−2.07, 0.55)	0.247	−0.03 (−0.81, 0.76)	0.946
2-isopropyl-4-methyl-pyrimidinol, ng/mL ^c^				
Tertile 2	−2.49 (−8.81, 3.83)	0.427	−1.64 (−5.04, 1.75)	0.332
Tertile 3	−2.10 (−6.78, 2.59)	0.368	**−3.85 (−7.25, −0.46)**	**0.027**
Log-transformed	−1.03 (−2.89, 0.84)	0.270	**−2.07 (−3.56, −0.58)**	**0.008**
Para-Nitrophenol, μg/L ^c^				
Tertile 2	−3.05 (−9.52, 3.42)	0.343	**−4.01 (−7.77, −0.25)**	**0.004**
Tertile 3	**−7.25 (−11.25, −3.25)**	**0.001**	**−3.73 (−6.89, −0.56)**	**0.023**
Log-transformed	−2.59 (−5.38, 0.20)	0.068	**−1.87 (−3.09, −0.64)**	**0.004**
Trans-dichlorovinyl-dimethylcyclopropane carboxylic acid, μg/L ^c^				
Tertile 2	2.00 (−6.33, 10.33)	0.628	0.53 (−3.38, 4.43)	0.785
Tertile 3	−1.89 (−7.46, 3.69)	0.495	−1.42 (−6.29, 3.44)	0.555
Log-transformed	−0.74 (−2.44, 0.96)	0.383	−0.55 (−2.15, 1.06)	0.493

aBeta, adjusted β coefficient; CI, confidence interval; DEET, N,N-Diethyl-meta-toluamide; Ref, reference. Variables with a value of *p* < 0.05 are shown in bold. ^a^ Adjusted for variables with a value of *p* < 0.05 in Supplementary Table S1, including age (continuous), BMI, hypertension, CVD, CKD, arthritis, and energy intake (continuous). ^b^ Adjusted for variables with a value of *p* < 0.05 in Supplementary Table S1, including age (continuous), race, BMI, hypertension, CVD, CKD, cancer, arthritis, energy intake (continuous), and protein consumption (categorical). ^c^ Tertile1 is a reference value for categorical variables.

## Data Availability

The data presented in this study are available on request from the corresponding author. The data are not publicly available due to restrictions concerning privacy and ethical reasons.

## Supplementary Materials

The following supporting information can be downloaded at: https://www.mdpi.com/article/10.3390/biomedicines12071461/s1. Supplementary Table S1. Characteristics of the study population (full). Supplementary Table S2. Distribution of urine pesticide levels (ng/mL creatinine) in adults with diabetes. Supplementary Table S3. Associations between study variables and muscle strength in adults with diabetes, stratified by sex. Supplementary Table S4. Association between urine pesticide levels and muscle strength in adults with diabetes, with vigorous intensity/moderate intensity, stratified by sex. Supplementary Table S5. Association between urine pesticide levels and muscle strength in adults with diabetes, with low intensity, stratified by sex. Supplementary Table S6. Association between urine pesticide levels and muscle strength in adults without diabetes, stratified by sex.
