# Supplementary material for: Associations between DEET, Organophosphorus Insecticides, and Handgrip Strength in Diabetes: An NHANES Analysis
Source: Biomedicines. 2024 Jul 1;12(7):1461. doi: 10.3390/biomedicines12071461 (PMC11274853; doi:10.3390/biomedicines12071461)
Supplement: Supplementary file 1 [file biomedicines-12-01461-s001.zip › biomedicines-3025011-supplementary.pdf]

Supplementary Table S1. Characteristics of the study population (full).

	Total	Male	Female	
	N = 412	n = 211	n = 201	P
**Muscle strength, kg**	67.4 ± 1.4	83.0 ± 1.5	50.8 ± 1.1	**<0.001**
**Age, years**	58.8 ± 0.9	58.9 ± 1.1	58.7 ± 1.2	0.885
< 50	99 (26.0)	50 (25.2)	49 (26.9)	0.302
50-59	83 (21.8)	41 (20.4)	42 (23.2)	
60-69	114 (26.1)	61 (30.7)	53 (21.1)	
70+	116 (26.1)	59 (23.7)	57 (28.7)	
**Race**				
Non-Hispanic White	154 (61.1)	82 (62.3)	72 (59.8)	0.646
Non-Hispanic Black	119 (15.8)	56 (13.6)	63 (18.0)	
Mexican American	52 (8.8)	29 (8.9)	23 (8.7)	
Others	87 (14.3)	44 (15.1)	43 (13.5)	
**Poverty-income ratio**				
Not poor, ≥ 1	274 (75.9)	145 (78.8)	129 (72.9)	0.158
Poor, < 1	138 (24.1)	66 (21.2)	72 (27.1)	
**Education level**				
High school and above	306 (81.0)	162 (82.1)	144 (79.8)	0.574
Never attended high school	106 (19.0)	49 (17.9)	57 (20.2)	
**Physical activity at leisure time METs-hours/week**				
Vigorous intensity	7 (1.2)	5 (1.7)	2 (0.6)	**<0.001**

Moderate intensity	78 (17.4)	24 (7.4)	54 (27.9)	
Low intensity	327 (81.4)	182 (90.8)	145 (71.5)	
**BMI, kg/m²**				
Normal (< 25)	50 (9.7)	25 (9.4)	25 (10.1)	0.980
Overweight (25-29.9)	131 (29.8)	74 (30.0)	57 (29.5)	
Obese (≥ 30.0)	231 (60.5)	112 (60.5)	119 (60.4)	
**Smoking status**				
Never	212 (50.1)	86 (37.1)	126 (63.9)	**<0.001**
Former	129 (32.5)	85 (43.1)	44 (21.3)	
Current	70 (17.4)	39 (19.8)	31 (14.8)	
Missing	1	1	0	
**Major comorbidity**				
Hypertension	290 (70.2)	142 (65.6)	148 (75.0)	0.113
Hyperlipidemia	312 (76.8)	152 (70.7)	160 (83.4)	**0.002**
CVD	106 (27.9)	60 (33.5)	46 (21.9)	0.051
COPD	38 (11.5)	11 (8.7)	27 (14.4)	0.275
CKD	85 (20.5)	41 (22.1)	44 (18.9)	0.568
Cancer	63 (19.5)	32 (22.7)	31 (16.1)	0.119
Arthritis	182 (42.8)	72 (32.4)	110 (53.8)	**0.002**
**Energy intake, kcal/day**	1990.3 ± 64.0	2294.6 ± 105.5	1667.2 ± 50.8	**<0.001**
Tertile 1 (< 1443.0)	137 (29.2)	46 (19.1)	91 (39.9)	**<0.001**
Tertile 2 (1443.0-2123.0)	138 (34.2)	66 (31.5)	72 (37.0)	
Tertile 3 (≥ 2123.0)	137 (36.7)	99 (49.4)	38 (23.2)	

**Protein consumption, g/day**	78.9 ± 2.6	90.9 ± 3.6	66.2 ± 3.0	**<0.001**
Tertile 1 (< 57.6)	137 (30.7)	45 (20.3)	92 (41.7)	**<0.001**
Tertile 2 (57.6-87.0)	138 (34.2)	71 (31.7)	67 (36.9)	
Tertile 3 (≥ 87.0)	137 (35.1)	95 (48.0)	42 (21.5)	
**Poor control of T2DM**	68 (16.5)	33 (15.5)	35 (17.5)	0.610
**Insulin dependence**	87 (21.7)	39 (19.4)	48 (24.2)	0.400

BMI, body mass index; CVD, cardiovascular disease; COPD, chronic obstructive pulmonary disease; CKD, chronic kidney disease; MET, metabolic equivalent task; T2DM, type 2 diabetes mellitus. Continuous variables are presented as mean ± standard error; categorical variables are presented as unweighted counts (weighted percentage). Variables with a value of P < 0.05 are shown in bold.

Supplementary Table S2. Distribution of urine pesticide levels (ng/mL creatinine) in adults with diabetes.

Pesticide	≥ LOD, % [a]	GM (95% CI)	Tertile 1	Tertile 2	Tertile 3
**DEET, ng/mL**	100.0	0.001 (0.0006-0.0015)	≤ 0.0004	0.0004-0.0008	> 0.0008
**DEET acid, ng/mL**	99.8	0.142 (0.0304-0.2530)	≤ 0.0087	0.0087-0.0282	> 0.0282
**Desethyl hydroxy DEET, ng/mL**	100.0	0.002 (0.0006-0.0033)	≤ 0.0005	0.0005-0.0009	> 0.0009
**2,4-dicholorphenoxyacetic acid, ng/ml**	100.0	0.006 (0.0044-0.0069)	≤ 0.0021	0.0021-0.0043	> 0.0043
**4-fluoro-3-phenoxy-benzoic acid, ng/ml**	100.0	0.003 (0.0019-0.0044)	≤ 0.0006	0.0006-0.0011	> 0.0011
**3-phenoxybenzoic acid, ng/ml**	99.8	0.025 (0.0144-0.0358)	≤ 0.0044	0.0044-0.0100	> 0.0100
**2-isopropyl-4-methyl-pyrimidinol, ng/ml**	100.0	0.003 (0.0014-0.0047)	≤ 0.0007	0.0007-0.0013	> 0.0013
***Para*-Nitrophenol, ug/L**	99.8	0.010 (0.0089-0.0118)	≤ 0.0046	0.0046-0.0088	> 0.0088
***Trans*-dichlorovinyl-dimethylcyclopropane carboxylic acid, ug/L**	100.0	0.024 (0.0080-0.0398)	≤ 0.0035	0.0035-0.0069	> 0.0069

DM, diabetes mellitus; LOD, limit of detection; GM, geometric mean; CI, confidence interval; DEET, N,N-Diethyl-meta-toluamide.

[a] Unweighted proportions.

Supplementary Table S3. Associations between study variables and muscle strength in adults with diabetes, stratified by sex

	Muscle strength, kg			
	Male		Female	
	n = 211	P	n = 201	P
**Age, years**	-5.23	**<0.001**	-6.96	**<0.001**
< 50	94.45 ± 3.84	**<0.001**	58.76 ± 1.17	**<0.001**
50-59	84.81 ± 1.93		53.24 ± 1.67	
60-69	81.20 ± 2.36		48.61 ± 2.19	
70+	71.69 ± 1.53		43.09 ± 1.24	
**Race**				
Non-Hispanic White	82.10 ± 2.39	0.120	49.34 ± 1.39	**0.012**
Non-Hispanic Black	89.71 ± 1.66		57.22 ± 1.50	
Mexican American	85.84 ± 1.03		50.45 ± 1.17	
Others	79.13 ± 2.35		49.14 ± 1.24	
**Poverty income ratio**				
Not poor, ≥ 1	83.18 ± 1.64	0.806	51.62 ± 1.31	0.176
Poor, < 1	82.47 ± 2.49		48.72 ± 1.42	
**Education level**				
High school and above	82.18 ± 1.21	0.414	51.46 ± 1.23	0.236
Never attended high school	86.90 ± 5.13		48.33 ± 2.16	
**Physical activity at leisure time, METs-hours/week**				
Men/women: ≥ 48.1 / 27.1	92.17	0.230	51.17	0.093
Men/women: 8.1 to ≤ 48 / 3.1 to ≤ 27	81.01 ± 0.97		53.29 ± 1.30	

Men/women: ≤ 8 / ≤ 3	83.02 ± 1.69		49.87 ± 1.29	
**BMI, kg/m^2**				
Normal (18.5-24.9)	70.91 ± 1.83	**<0.001**	46.73 ± 0.79	**0.007**
Overweight (25-29.9)	85.45 ± 1.86		47.22 ± 1.29	
Obese (≥ 30.0)	83.71 ± 1.87		53.27 ± 1.65	
**Smoking status**				
Never	82.77 ± 1.64	0.707	51.11 ± 1.39	0.424
Former	82.06 ± 3.16		48.96 ± 1.09	
Current	85.31 ± 2.31		52.29 ± 1.88	
**Excessive alcohol consumption**	80.63 ± 3.16	0.529	53.13	0.666
**Major comorbidity**				
Hypertension	80.20 ± 1.50	**0.008**	49.89 ± 1.26	**0.045**
Hyperlipidemia	82.03 ± 1.11	0.510	50.07 ± 1.15	0.068
CVD	73.16 ± 2.03	**<0.001**	44.37 ± 0.85	**<0.001**
COPD	77.26	0.185	51.67 ± 0.36	0.712
CKD	75.45 ± 2.92	**0.011**	46.11 ± 0.91	**0.005**
Cancer	80.22 ± 1.05	0.088	42.35 ± 2.05	**0.002**
Arthritis	77.71 ± 2.14	**0.021**	46.65 ± 1.25	**<0.001**
Energy intake, kcal/day	4.20	**<0.001**	2.79	**0.009**
Tertile 1	75.52 ± 3.09	**0.005**	47.18 ± 1.13	**0.002**
Tertile 2	79.74 ± 1.48		51.75 ± 1.49	
Tertile 3	88.02 ± 2.48		55.64 ± 1.46	
Protein consumption, g/day	1.76	0.089	1.06	0.296

Tertile 1	78.54 ± 1.77	0.096	47.68 ± 1.08	**0.023**
Tertile 2	82.09 ± 3.85		52.94 ± 1.45	
Tertile 3	85.54 ± 1.77		53.33 ± 1.22	
Poor control of diabetes	83.89 ± 2.81	0.801	52.93 ± 0.63	0.277
Insulin dependence	80.82 ± 0.67	0.315	48.85 ± 2.11	0.294

BMI, body mass index; CVD, cardiovascular disease; COPD, chronic obstructive pulmonary disease; CKD, chronic kidney disease; MET, metabolic equivalent task.

Variables with a value of P < 0.05 are shown in bold.

Supplementary Table S4. Association between urine pesticide levels and muscle strength in adults with diabetes, with vigorous intensity/moderate intensity, stratified by sex.

	**Muscle strength**			
	**Male [a]**		**Female [b]**	
**Pesticide**	aBeta (95% CI)	p-value	aBeta (95% CI)	p-value
**DEET, ng/mL [c]**				
Tertile2	**-13.18 (-22.12, -4.24)**	**0.006**	1.39 (-9.46,12.23)	0.795
Tertile3	**-11.73 (-23.32, -0.14)**	**0.048**	-5.99 (-14.66,2.68)	0.169
Log-transformed	**-6.67 (-12.97, -0.36)**	**0.039**	-3.02 (-7.29,1.25)	0.158
**DEET acid, ng/mL [c]**				
Tertile2	0.64 (-11.44,12.72)	0.913	0.80 (-5.19,6.79)	0.786
Tertile3	-8.24 (-20.24,3.77)	0.168	4.21 (-1.53,9.96)	0.144

Log-transformed	**-2.64 (-4.60, -0.68)**	**0.011**	0.49 (-0.78,1.76)	0.438
**Desethyl hydroxy DEET, ng/mL [c]**				
Tertile2	**-13.27 (-22.20, -4.34)**	**0.006**	0.14 (-10.52,10.80)	0.979
Tertile3	-9.53 (-26.51,7.45)	0.255	-5.65 (-14.59,3.30)	0.207
Log-transformed	**-4.98 (-9.19, -0.76)**	**0.023**	-1.50 (-5.38,2.39)	0.437
**2,4-dicholorphenoxyacetic acid, ng/ml [c]**				
Tertile2	-11.20 (-23.00,0.60)	0.062	-2.44 (-11.02,6.15)	0.566
Tertile3	1.08 (-11.53,13.69)	0.860	-4.53 (-9.36,0.31)	0.065
Log-transformed	2.51 (-3.71,8.72)	0.411	-0.41 (-2.54,1.72)	0.697
**4-fluoro-3-phenoxy-benzoic acid, ng/ml [c]**				
Tertile2	-2.13 (-16.97,12.72)	0.768	-0.56 (-11.13,10.00)	0.914
Tertile3	-4.94 (-16.51,6.63)	0.384	-2.04 (-8.35,4.28)	0.515
Log-transformed	-0.51 (-4.19,3.18)	0.778	-0.49 (-3.01,2.04)	0.696
**3-phenoxybenzoic acid, ng/ml [c]**				
Tertile2	-6.03 (-19.83,7.77)	0.373	-0.02 (-6.81,6.76)	0.995
Tertile3	3.06 (-13.64,19.76)	0.706	-2.36 (-7.53,2.81)	0.358
Log-transformed	0.09 (-4.34,4.52)	0.965	-0.87 (-2.45,0.71)	0.271
**2-isopropyl-4-methyl-pyrimidinol, ng/ml [c]**				
Tertile2	1.79 (-12.24,15.82)	0.793	-3.98 (-10.26,2.30)	0.205
Tertile3	5.75 (-4.41,15.91)	0.252	-4.40 (-10.93,2.12)	0.178
Log-transformed	-0.59 (-4.33,3.15)	0.745	**-2.17 (-4.22, -0.11)**	**0.039**
***Para*-Nitrophenol, ug/L [c]**				
Tertile2	-9.55 (-22.25,3.15)	0.132	-3.89 (-10.14,2.35)	0.212

Tertile3	1.11 (-9.39,11.60)	0.828	**-8.44 (-12.88, -3.99)**	**<0.001**
Log-transformed	-1.65 (-6.84,3.54)	0.515	-2.84 (-6.52,0.84)	0.125
***Trans*-dichlorovinyl-dimethylcyclopropane carboxylic acid, ug/L** [c]				
Tertile2	-9.43 (-22.88,4.02)	0.159	0.83 (-9.44,11.10)	0.870
Tertile3	-6.30 (-19.61,7.02)	0.336	-2.39 (-9.45,4.66)	0.494
Log-transformed	-2.54 (-5.30,0.22)	0.069	-0.70 (-3.10,1.70)	0.555

aBeta, adjusted β coefficient; BMI, body mass index; CI, confidence interval; CKD, chronic kidney disease; CVD, cardiovascular disease; DEET, N,N-Diethyl-meta-toluamide; Ref, reference. Variables with a value of P < 0.05 are shown in bold. [a] Adjusted for variables with a value of P < 0.05 in Supplementary Table 1, including age (continuous), BMI, hypertension, CVD, CKD, arthritis, and energy intake (continuous). [b] Adjusted for variables with a value of P < 0.05 in Supplementary Table 1, including age (continuous), race, BMI, hypertension, CVD, CKD, cancer, arthritis, energy intake (continuous), and protein consumption (categorical). [c] Tertile1 is reference value for categorical variable.

Supplementary Table S5. Association between urine pesticide levels and muscle strength in adults with diabetes, with low intensity, stratified by sex.

	Muscle strength			
	**Male** [a]		**Female** [b]	
**Pesticide**	aBeta (95% CI)	p-value	aBeta (95% CI)	p-value
**DEET, ng/mL** [c]				
Tertile2	3.08 (-2.72,8.87)	0.284	-1.41 (-7.24,4.42)	0.624
Tertile3	-3.87 (-15.27,7.53)	0.490	-1.14 (-4.97,4,2.69)	0.547
Log-transformed	**-7.33 (-13.48, -1.19)**	**0.021**	-0.42 (-3.14,2.30)	0.755
**DEET acid, ng/mL** [c]				
Tertile2	-0.16 (-7.18,6.87)	0.964	0.19 (-3.43,3.82)	0.915

Tertile3	-0.08 (-7.42,7.26)	0.983	-1.11 (-5.72,3.49)	0.625
Log-transformed	-1.13 (-3.10,0.83)	0.245	-0.08 (-1.51,1.36)	0.914
**Desethyl hydroxy DEET, ng/mL** [c]				
Tertile2	3.65 (-1.97,9.27)	0.193	-1.47 (-7.79,4.84)	0.637
Tertile3	-4.74 (-14.98,5.50)	0.349	-1.54 (-4.77,1.68)	0.337
Log-transformed	**-7.15 (-12.14, -2.16)**	**0.007**	-0.42 (-2.86,2.02)	0.729
**2,4-dicholorphenoxyacetic acid, ng/ml** [c]				
Tertile2	-3.10 (-8.79,2.59)	0.273	-1.39 (-5.30,2.53)	0.475
Tertile3	-4.57 (-14.03,4.90)	0.330	-1.26 (-6.02,3.49)	0.592
Log-transformed	-1.33 (-4.48,1.81)	0.390	0.15 (-1.89,2.20)	0.879
**4-fluoro-3-phenoxy-benzoic acid, ng/ml** [c]				
Tertile2	0.02 (-7.30,7.35)	0.995	-3.62 (-9.20,1.96)	0.195
Tertile3	-3.24 (-16.94,10.47)	0.630	-1.47 (-5.00,2.06)	0.402
Log-transformed	-4.05 (-9.11,1.01)	0.111	-0.13 (-1.65,1.39)	0.861
**3-phenoxybenzoic acid, ng/ml** [c]				
Tertile2	-0.13 (-6.47,6.20)	0.966	-0.27 (-4.37,3.84)	0.895
Tertile3	**-7.01 (-13.85, -0.17)**	**0.045**	0.84 (-1.96,3.63)	0.545
Log-transformed	-2.09 (-4.42,0.23)	0.076	0.27 (-0.72,1.27)	0.576
**2-isopropyl-4-methyl-pyrimidinol, ng/ml** [c]				
Tertile2	-0.91 (-7.78,5.95)	0.786	-2.08 (-6.17,2.00)	0.306
Tertile3	-9.01 (-20.49,2.47)	0.118	**-3.21 (-6.07, -0.35)**	**0.029**
Log-transformed	**-6.07 (-11.47, -0.66)**	**0.029**	**-1.89 (-3.11, -0.67)**	**0.004**
***Para*-Nitrophenol, ug/L** [c]				

Tertile2	-5.30 (-14.18,3.59)	0.231	-3.78 (-8.44,0.88)	0.108
Tertile3	0.25 (-9.11,9.61)	0.957	-3.76 (-8.18,0.65)	0.092
Log-transformed	-3.01 (-8.75,2.74)	0.291	**-1.52 (-3.02, -0.02)**	**0.047**
***Trans*-dichlorovinyl-dimethylcyclopropane carboxylic acid, ug/L [c]**				
Tertile2	0.31 (-5.60,6.23)	0.914	0.05 (-4.49,4.60)	0.981
Tertile3	-10.69 (-21.39,0.01)	0.050	-0.24 (-5.15,4.67)	0.922
Log-transformed	**-6.95 (-12.80, -1.10)**	**0.022**	0.01 (-1.60,1.63)	0.987

aBeta, adjusted β coefficient; BMI, body mass index; CI, confidence interval; CKD, chronic kidney disease; CVD, cardiovascular disease; DEET, N,N-Diethyl-meta-toluamide; Ref, reference. Variables with a value of P < 0.05 are shown in bold. [a] Adjusted for variables with a value of P < 0.05 in Supplementary Table 1, including age (continuous), BMI, hypertension, CVD, CKD, arthritis, and energy intake (continuous). [b] Adjusted for variables with a value of P < 0.05 in Supplementary Table 1, including age (continuous), race, BMI, hypertension, CVD, CKD, cancer, arthritis, energy intake (continuous), and protein consumption (categorical). [c] Tertile1 is reference value for categorical variable.

Supplementary Table S6. Association between urine pesticide levels and muscle strength in adults without diabetes, stratified by sex.

	**Muscle strength**			
	**Male [a] (n=1061)**		**Female [b] (n=1034)**	
Phenols and parabens	aBeta (95% CI)	p-value	aBeta (95% CI)	p-value
**DEET, ng/mL**				
Log-transformed	-0.19 (-1.46,1.08)	0.764	0.14 (-0.78,1.06)	0.763
**DEET acid, ng/mL**				
Log-transformed	0.40 (-0.45,1.24)	0.346	-0.28 (-0.77,0.21)	0.251
**Desethyl hydroxy DEET, ng/mL**				

Log-transformed	0.08 (-1.03,1.18)	0.888	0.11 (-0.58,0.79)	0.749
**2,4-dicholorphenoxyacetic acid, ng/ml**				
Log-transformed	1.82 (-0.12,3.75)	0.065	-0.19 (-1.38,1.00)	0.749
**4-fluoro-3-phenoxy-benzoic acid, ng/ml**				
Log-transformed	-0.34 (-1.79,1.10)	0.632	-0.63 (-1.83,0.57)	0.294
**3-phenoxybenzoic acid, ng/ml**				
Log-transformed	-1.00 (-2.05,0.04)	0.059	-0.57 (-1.21,0.07)	0.078
**2-isopropyl-4-methyl-pyrimidinol, ng/ml**				
Log-transformed	-0.71 (-1.71,0.29)	0.158	-0.18 (-1.31,0.94)	0.740
***Para*-Nitrophenol, ug/L**				
Log-transformed	-1.10 (-2.81,0.61)	0.201	-0.40 (-1.18,0.37)	0.293
***Trans*-dichlorovinyl-dimethylcyclopropane carboxylic acid, ug/L**				
Log-transformed	-0.23 (-1.46,1.00)	0.703	-0.14 (-1.00,0.71)	0.733

aBeta, adjusted β coefficient; BMI, body mass index; CI, confidence interval; CKD, chronic kidney disease; CVD, cardiovascular disease; DEET, N,N-Diethyl-meta-toluamide; Ref, reference.

Variables with a value of $P < 0.05$ are shown in bold.

[a] Adjusted for including age (continuous), BMI, hypertension, CVD, CKD, arthritis, and energy intake (continuous).

[b] Adjusted for age (continuous), race, BMI, hypertension, CVD, CKD, cancer, arthritis, energy intake (continuous), and protein consumption (categorical).
